# Supplementary material for: Impact of COVID-19 pandemic on utilization of essential maternal healthcare services in Ethiopia: A systematic review and meta-analysis
Source: PLoS One. 2023 Feb 7;18(2):e0281260. doi: 10.1371/journal.pone.0281260 (PMC9904479; doi:10.1371/journal.pone.0281260)
Supplement: S2 Table — (DOCX) [file pone.0281260.s002.docx]

S2 Table: The Newcastle-Ottawa scale for quality assessment of primary studies

| **Studies ID** | **Selection**  **(Maximum of five star)** | **Comparability**  **(Maximum two star)** | **Outcome assessment**  **(Maximum of three stars)** | **Overall quality** |
| --- | --- | --- | --- | --- |
| Ayele et al. | ******** | ****** | ******* | 9 |
| Shimels T. | ******** | ****** | ****** | 8 |
| Dandena et al. | ******** | ****** | ****** | 8 |
| Kassie et al. | ******** | ****** | ******* | 9 |
| Abdela et al. | ******** | ***** | ****** | 7 |
| Tolu et al. | ******* | ***** | ******* | 7 |
| Gebreegziabher et al. | ******** | ***** | ****** | 8 |
| Bantalem et al. | ******* | ***** | ****** | 6 |
| Shuka et al. | ******** | ***** | ******* | 8 |
| Desta et al. | ******** | ****** | ******* | 9 |
| Oladeji et al. | ******* | ***** | ****** | 6 |
| Tadesse E. | ******** | ****** | ******* | 9 |
| Temesgen et al. | ******** | ****** | ******* | 9 |
| Seme A et al. | ******* | ***** | ****** | 6 |
| Enbiale et al. | ******** | ****** | ****** | 8 |
| UNICEF | ******** | ***** | ****** | 7 |
| Workicho et al. | ******** | ***** | ******* | 8 |
| Hailemariam et al. | NA | NA | NA | NA |
| Zimmerman et al. | ******** | ***** | ******* | 8 |
| Temesgen et al. | ******** | ****** | ******* | 9 |
| Tilahun et al. | ******** | ***** | ****** | 7 |

NA: not assessed
